# Dataset on effects of perinatal exposure to propylthiouracil on serum T4, body weight gain, day of eye opening and brain monoamine contents in offspring mice

**DOI:** 10.1016/j.dib.2019.104900

**Published:** 2019-11-29

**Authors:** Toyoshi Umezu, Taizo Kita, Masatoshi Morita

**Affiliations:** a Center for Health and Environmental Risk Research, National Institute for Environmental Studies, Tsukuba, Ibaraki, 305-8506, Japan; b Graduate School of Food and Nutrition, Kyushu Nutrition Welfare University, Kitakyushu, Fukuoka, 803-8511, Japan; c Graduate School of Agriculture, Ehime University, Matsuyama, Ehime, 790-8577, Japan

**Keywords:** Hypothyroidism, Growth, Neurodevelopment, Monoaminergic neurotransmitter, Mouse

## Abstract

Physical growth and brain development need sufficient thyroid hormone (TH). This dataset describes serum T4 level, body weight gain and day of eye opening in offspring mice exposed to the TH synthesis inhibitor propylthiouracil (PTU) from gestational day (GD) 15 to postnatal day (PND) 25. This dataset also describes content of dopamine (DA), 3,4-dihydroxyphenylacetic acid (DOPAC), homovanillic acid (HVA), 3-methoxytyramine (3-MT), 5-HT, and 5-hydroxyindoleacetic acid (5-HIAA) and their turnover in the hypothalamus, hippocampus, and nucleus accumbens of male offspring mice perinatally exposed to PTU. These data are referred by a research article entitled “Hyperactive behavioral phenotypes and an altered brain monoaminergic state in male offspring mice with perinatal hypothyroidism” [1].

**Table:** Specifications Table

Subject	Toxicology
Specific subject area	Development of brain monoaminergic system and hypothyroidism
Type of data	Figures and Tables
How data were acquired	Observation and body weight measurement Radioimmunoassay using DPC・total T4 kits (Yatoron Co., Ltd. (present: LSI Medience Co., Ltd.), Tokyo, Japan) High performance liquid chromatography system (L-5000; Yanaco, Kyoto, Japan) with a 4.6 mm × 250 mm ODS-C18 column (Nacalai, Kyoto, Japan) and an electrochemical detector (VMD-101A; Yanaco) including a glassy carbon electrode.
Data format	Raw
Parameters for data collection	125, 250, or 500 ppm PTU solution was administered to dams from GD 15 to PND25. Tap water was given to control dams. Tap water was given to all offspring mice thereafter. Every 3–4 days after delivery, the body weight and eye opening of offspring were checked. Blood samples for T4 radioimmunoassay were collected from dams and offspring on PND25. Brain samples for monoamine assay were obtained from male offspring on PND60.
Description of data collection	Body weight gain data was obtained by measuring every 3–4 days. The day of eye opening was determined by every 3–4 days observation. Serum T4 level was determined using radioimmunoassay. Monoamine contents in brain were measured using HPLC/ECD.
Data source location	Institution: National Institute for Environmental Studies City/Town/Region: Tsukuba, Ibaraki Country: Japan
Data accessibility	Data is provided with this article
Related research article	Author's name Umezu T., Kita T., Morita M. Title Hyperactive behavioral phenotypes and an altered brain monoaminergic state in male offspring mice with perinatal hypothyroidism Journal Toxicology Reports 6 (2019) 1031–1039 https://doi.org/10.1016/j.toxrep.2019.10.005

**Table:** 

**Value of the Data** • The data show that perinatal exposure to thyroid hormone synthesis inhibitor propylthiouracil retards growth and affects development of brain monoaminergic system in offspring mice. • Data could be valuable for researches on impact of perinatal hypothyroidism on brain monoaminergic system development. • Data could be useful for researchers to further study roles of thyroid hormone for development of brain monoaminergic system. • Data could be used by other researchers to study neurodevelopmental effects of chemicals that disrupt thyroid hormone system (thyroid hormone disruptors). • Data could be used by other researchers to study central mechanisms underlie mental problems produced by perinatal hypothyroidism.

## 1 Data

**Fig. 1:**
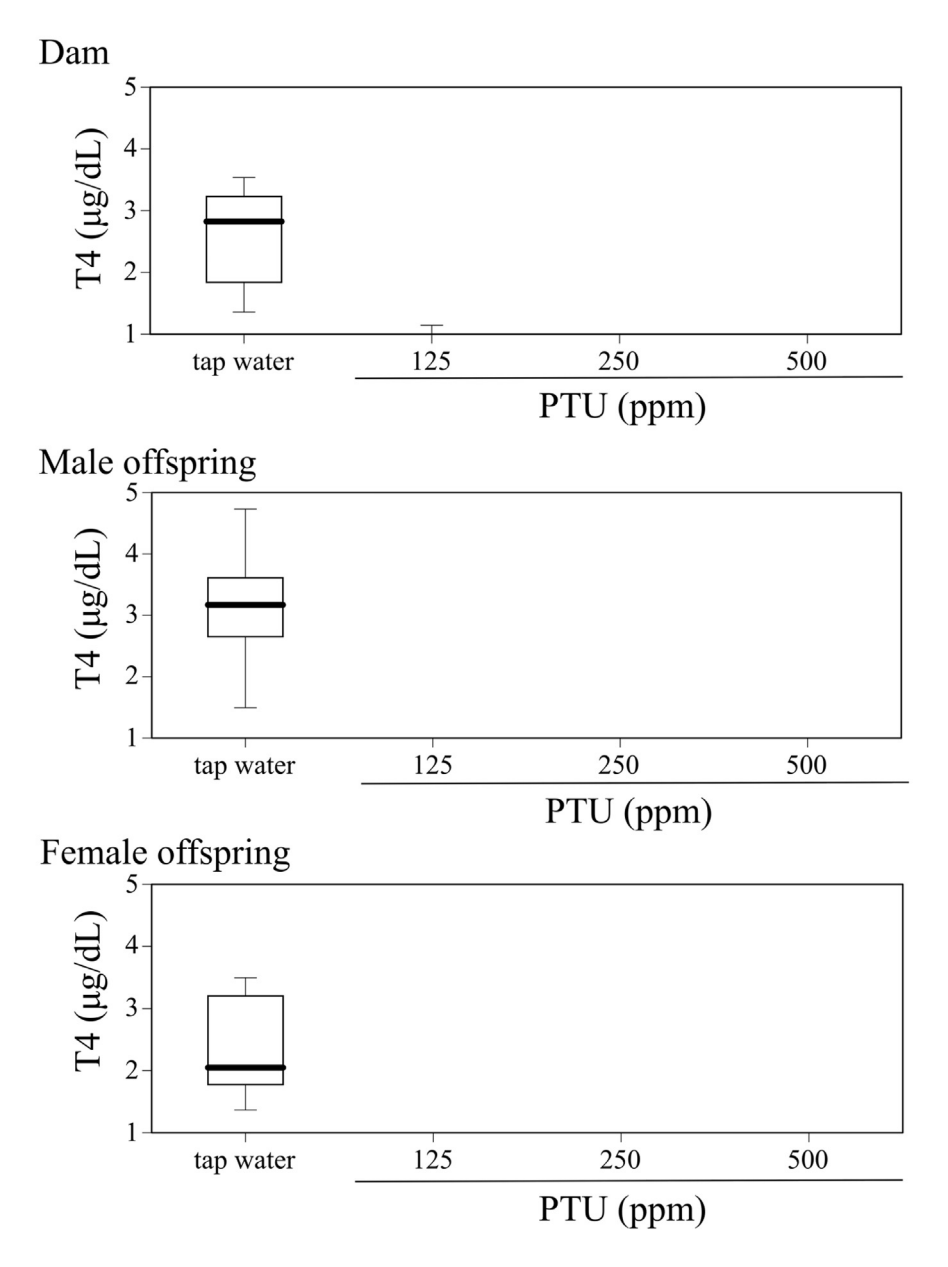
Serum T4 on PND 25 in dams (top panel), male offspring (middle panel), and female offspring (bottom panel) administered tap water or 125, 250, or 500 ppm PTU solution from GD 15 to PND 25. The limit of detection was 1.0 μg/dL. Data are shown using a box plot. N = 7–9 animals per group.

**Fig. 2:**
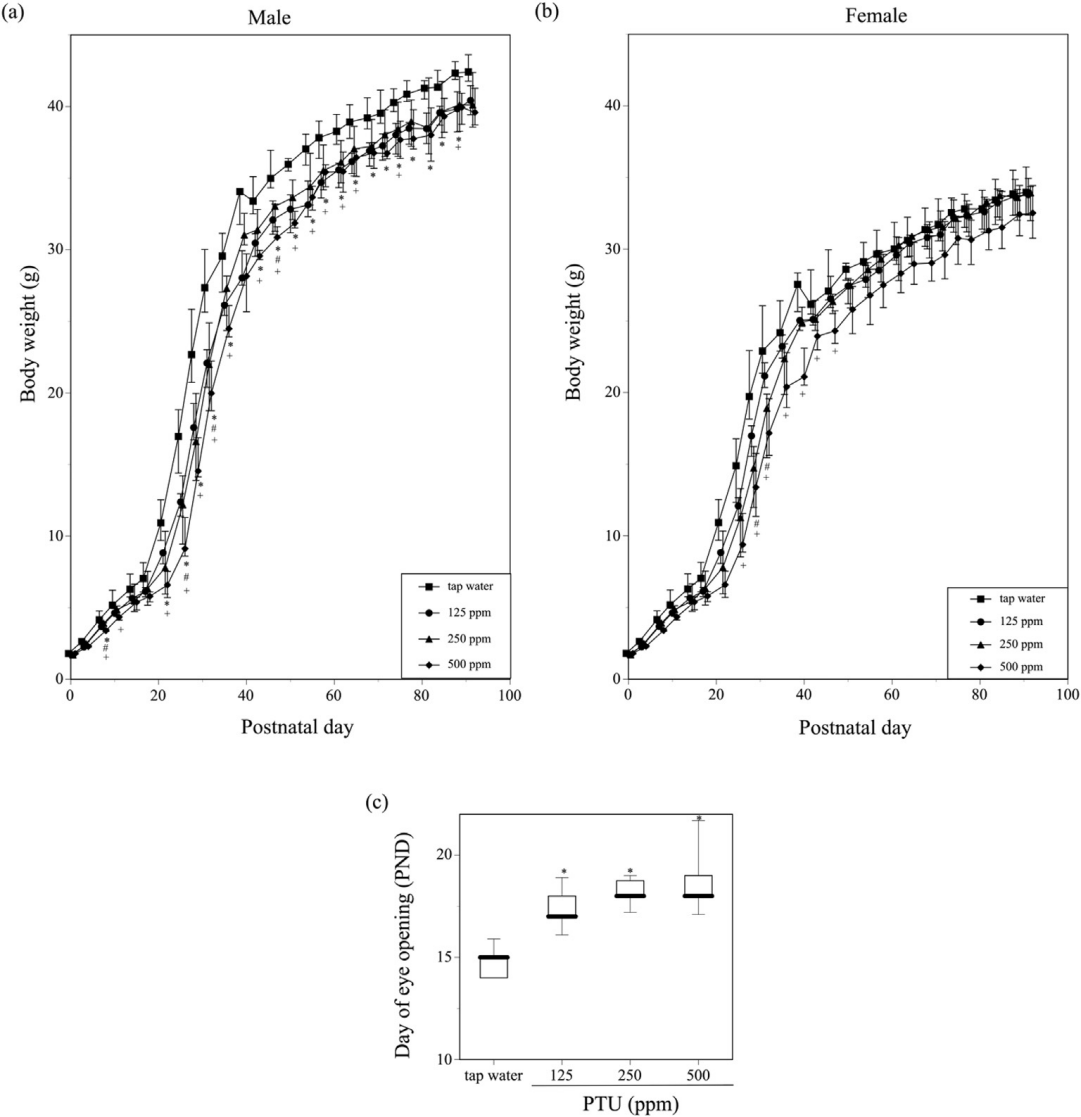
Alterations in body weight in (a) male and (b) female offspring mice after birth. Their dams were administered 125, 250, or 500 ppm PTU solution from GD 15 to PND 25. Tap water was given to the control dams during the same perinatal period. Body weights of all littermates in a litter were measured, and the average body weight was assigned as the body weight per pup. After PND25 when offspring were weaned from the dams, body weights of all littermates of the same gender in a litter were measured, and the average body weight was assigned as the body weight of that gender of offspring. Mean body weights are indicated by symbols; standard errors of the mean (SEM) are indicated by vertical lines. N = 6–7 litters per group. Water vs. 125 ppm; *P < 0.05, water vs. 250 ppm; #P < 0.05, water vs. 500 ppm; +P < 0.05. (c) The day when eye opening was observed in the offspring mice whose dams were given tap water or 125, 250, or 500 ppm PTU solution. *P < 0.05 vs. tap water. Data are shown using a box plot, in which thick black lines indicate median, boxes indicate the 1st and 3rd quartiles and vertical lines indicate maximum and minimum values.

**Fig. 3:**
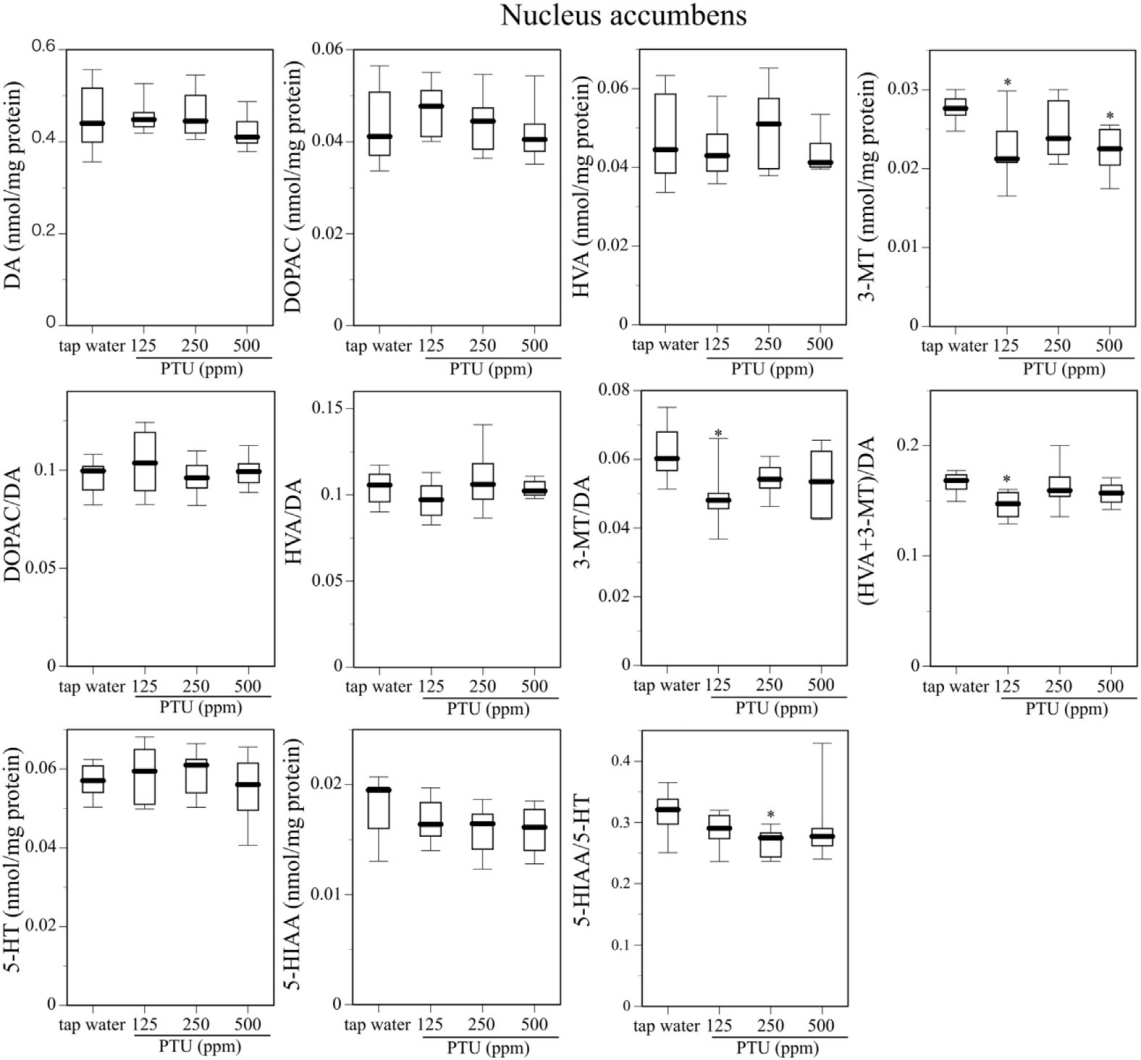
Content of DA and DA metabolites (DOPAC, HVA, 3-MT), contents of 5-HT and the 5-HT metabolite (5-HIAA), DA turnover (DOPAC/DA, HVA/DA, 3-MT/DA, (HVA+3-MT)/DA), and 5-HT turnover (5-HIAA/5-HT) in the nucleus accumbens of the male offspring mice given tap water or 125, 250, or 500 ppm PTU solution from GD15 to PND25. Data are shown using a box plot. *P < 0.05 compared with the tap water-administered control. N = 8 animals per group.

**Fig. 4:**
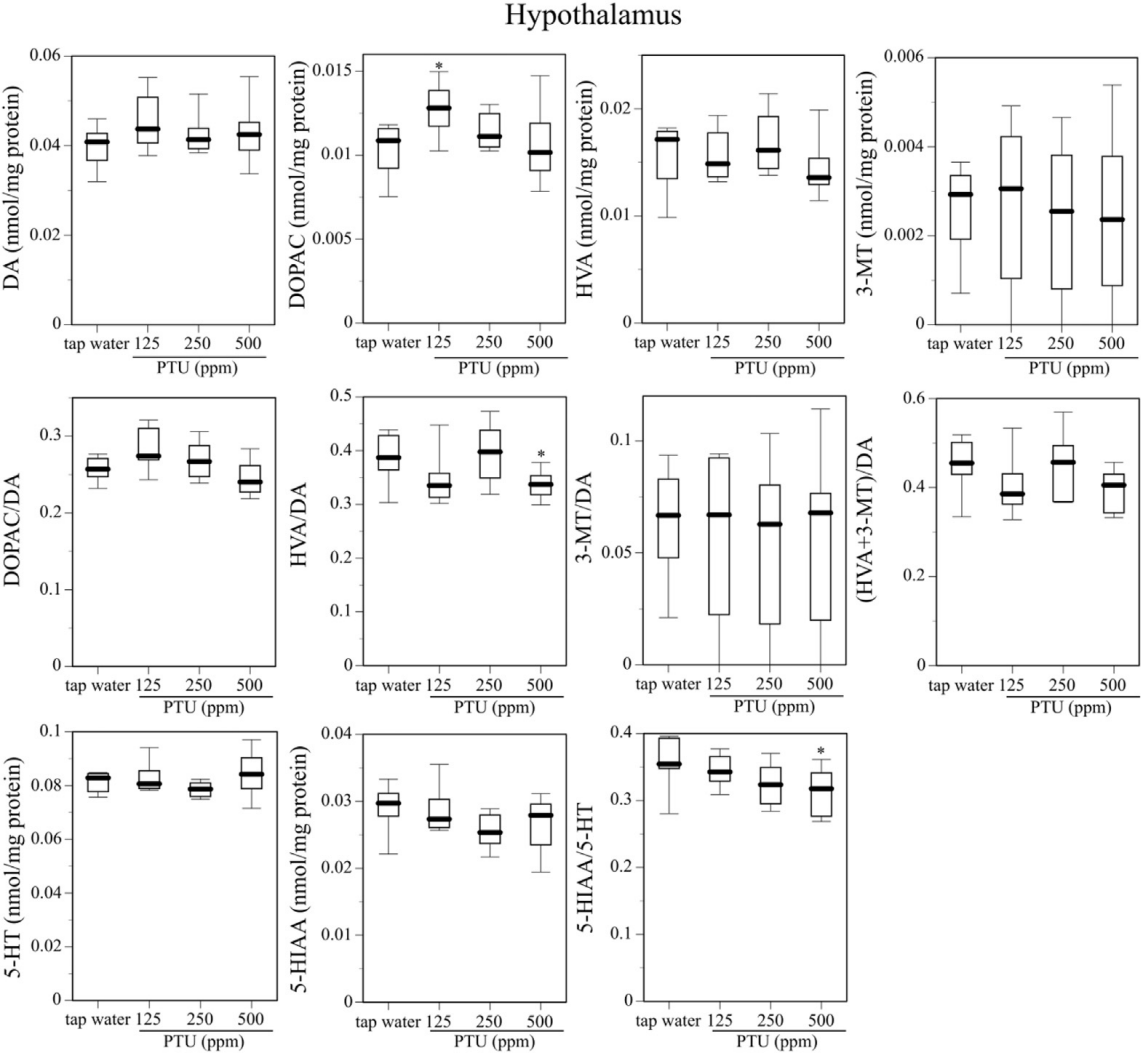
Contents of DA and DA metabolites, contents of 5-HT and the 5-HT metabolite, DA turnover, and 5-HT turnover in the hypothalamus of the male offspring mice given tap water or 125, 250, or 500 ppm PTU solution from GD15 to PND25. Data are shown similarly to Fig. 3.

**Fig. 5:**
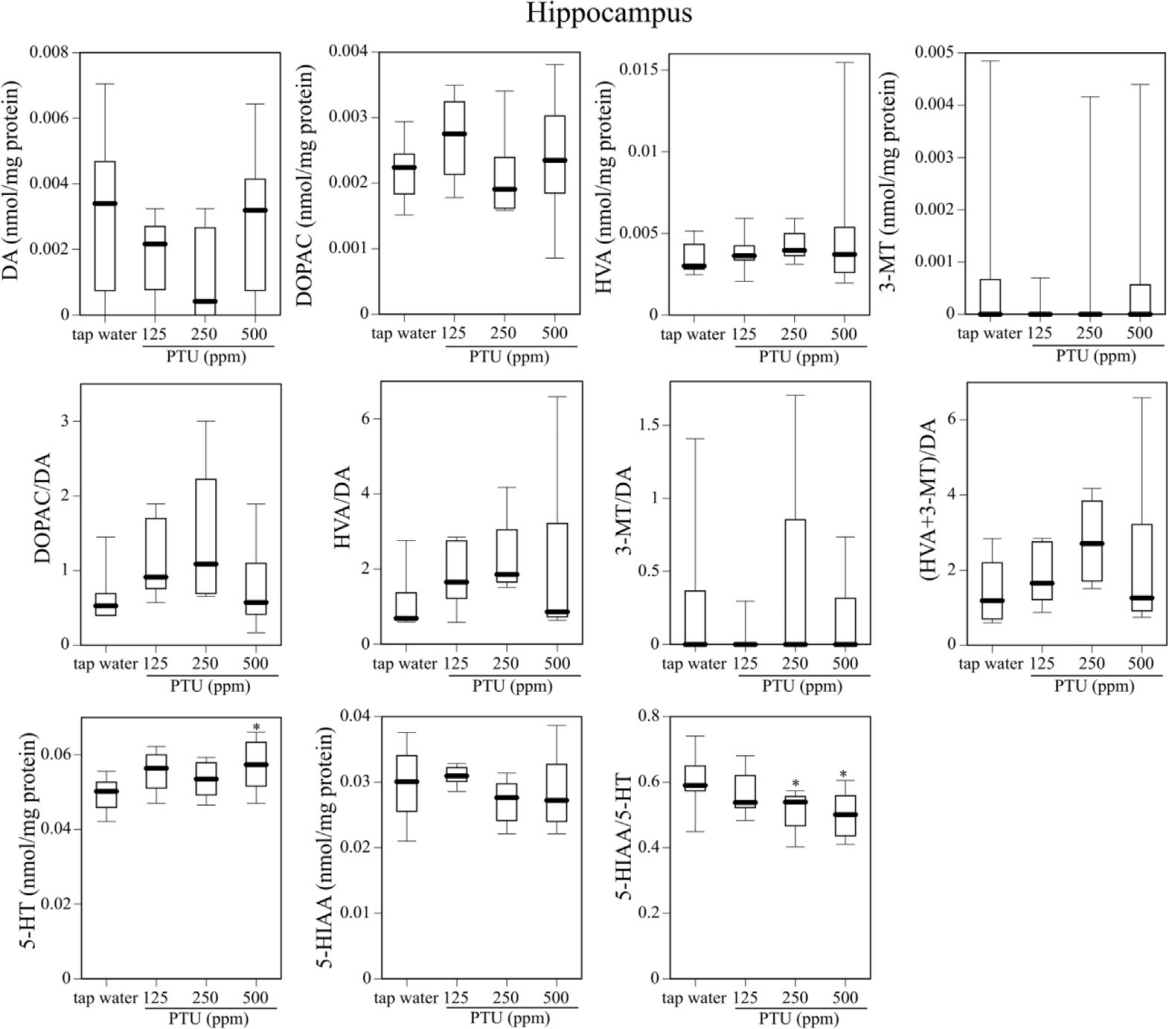
Contents of DA and DA metabolites, contents of 5-HT and the 5-HT metabolite, DA turnover, and 5-HT turnover in the hippocampus of the male offspring mice given tap water or 125, 250, or 500 ppm PTU solution from GD15 to PND25. Data are shown similarly to Fig. 3.

Fig. 1 describes serum T4 level on PND25 in dams, male and female offspring mice given tap water or 125, 250, or 500 ppm PTU solution from GD 15 to PND 25. Fig. 2 shows body weight gain and the day of eye opening in offspring mice given tap water or 125, 250, or 500 ppm PTU solution from GD 15 to PND. Fig. 3, Fig. 4 and Fig. 5 describes content of DA, DOPAC, HVA, 3-MT, 5-HT, and 5-HIAA and their turnover in the nucleus accumbens, hypothalamus, and hippocampus, respectively, of male offspring mice given tap water or 125, 250, or 500 ppm PTU solution from GD 15 to PND 25. Ambulatory activity and content of DA, DOPAC, HVA, 3-MT, 5-HT, and 5-HIAA and their turnover in the striatum of the same male offspring mice are described in the related article [1]. Row data for Figs. 3, Fig. 4, Fig. 5 are described in Table 1, Table 2 and Table 3, respectively.

**Table 1:** Data on content of DA and DA metabolites (DOPAC, HVA, 3-MT), contents of 5-HT and the 5-HT metabolite (5-HIAA), DA turnover (DOPAC/DA, HVA/DA, 3-MT/DA, (HVA+3-MT)/DA), and 5-HT turnover (5-HIAA/5-HT) in the nucleus accumbens of the male offspring mice given tap water or 125, 250, or 500 ppm PTU solution from GD15 to PND25. Medians, 1st quartiles and 3rd quartiles of these data are shown in Fig. 3. Unit of data is nmol/mg protein.

Sample Name	Dopamine	DOPAC	HVA	3MT	DOPAC/DA	HVA/DA	3-MT/DA	(HVA+3MT)/DA	5-HT	5-HIAA	5HIAA/5HT
Tap water											
NC-1	0.34231152	0.03553294	0.03245417	0.02654695	0.103802930	0.094808885	0.077552006	0.172360891	0.05350724	0.01278465	0.238933174
NC-5	0.499971651	0.049832939	0.059139657	0.03022973	0.099671530	0.11828602	0.060462893	0.178748912	0.05960127	0.01936303	0.324876102
NC-9	0.46717663	0.04131942	0.04533739	0.02801751	0.088444966	0.09704551	0.059971985	0.157017495	0.06261998	0.01984294	0.316878722
NC-13	0.410505137	0.04095695	0.043669769	0.02724353	0.099772076	0.106380566	0.066365879	0.172746446	0.061982572	0.01963842	0.316837842
NC-17	0.53321981	0.05856603	0.05813628	0.02953972	0.109834686	0.109028739	0.055398763	0.164427502	0.05456791	0.01842202	0.337597932
NC-21	0.38818994	0.03858072	0.04074922	0.02698555	0.099386190	0.104972386	0.069516344	0.17448873	0.05814687	0.01963660	0.337706822
NC-25	0.56701078	0.05176454	0.06518133	0.02812620	0.091293744	0.114956076	0.049604356	0.164560432	0.05592501	0.02106639	0.376690046
NC-29	0.41293468	0.03285000	0.03636900	0.02399236	0.079552536	0.08807446	0.058102066	0.146176526	0.04897408	0.01360646	0.277829828
PTU125PPM											
NC-2	0.42472257	0.04051011	0.03479736	0.03092939	0.095380167	0.081929623	0.072822578	0.154752200	0.06090494	0.01371133	0.225126674
NC-6	0.45254931	0.05056653	0.04516935	0.02087694	0.11173706	0.099810885	0.046131851	0.145942736	0.05798684	0.01651613	0.284825448
NC-10	0.54978222	0.04490893	0.06078043	0.02725702	0.081684952	0.110553647	0.049577843	0.160131490	0.06574069	0.02016520	0.306738576
NC-14	0.41534117	0.05096248	0.03825521	0.02069908	0.122700283	0.092105501	0.049836333	0.141941834	0.06412279	0.01861777	0.290345543
NC-18	0.45347442	0.05668014	0.05170151	0.02112808	0.124990826	0.114011959	0.046591561	0.160603520	0.04957267	0.01592773	0.321300624
NC-22	0.47308439	0.03985358	0.03984769	0.02135201	0.084242009	0.08422957	0.045133608	0.129363177	0.06914768	0.01811901	0.262033502
NC-26	0.44098243	0.04172470	0.04344520	0.02220201	0.094617609	0.098519108	0.050346706	0.148865814	0.05064037	0.01470252	0.290331921
NC-30	0.44383459	0.05126589	0.04252944	0.01473327	0.115506751	0.095822724	0.033195402	0.129018125	0.05147914	0.01626032	0.315862306
PTU250PPM											
NC-3	0.54893556	0.05773628	0.05822788	0.03061766	0.105178620	0.10607416	0.055776412	0.16185057	0.06143978	0.01717115	0.279479425
NC-7	0.42520042	0.04748261	0.05391980	0.02315738	0.111671134	0.12681031	0.054462273	0.18127258	0.06107815	0.01660169	0.271810678
NC-11	0.46535459	0.04515600	0.06823369	0.02859555	0.097035671	0.14662730	0.06144894	0.20807624	0.06343474	0.01917272	0.302243251
NC-15	0.53596422	0.04721323	0.05685163	0.02861155	0.088090256	0.10607355	0.053383313	0.159456859	0.06089468	0.01742916	0.286218116
NC-19	0.44007481	0.04378986	0.04816763	0.02195121	0.099505493	0.10945328	0.049880632	0.159333917	0.05178742	0.01438455	0.277761366
NC-23	0.44979707	0.03562581	0.03791626	0.02011308	0.079204191	0.08429636	0.044715888	0.129012251	0.06775599	0.01627364	0.240180075
NC-27	0.40156785	0.03814432	0.04141340	0.02165368	0.094988474	0.10312928	0.053922853	0.157052137	0.05608195	0.01386622	0.247249278
NC-31	0.41232012	0.03859665	0.03784339	0.02449439	0.093608459	0.09178157	0.059406234	0.151187804	0.04959836	0.01165253	0.234937871
PTU500PPM											
NC-4	0.50206590	0.05776744	0.05605222	0.02149964	0.115059483	0.11164314	0.04282234	0.154465487	0.05832924	0.01704379	0.292199791
NC-8	0.45177605	0.04083439	0.04461650	0.01939459	0.09038636	0.09875799	0.04292966	0.141687652	0.06099488	0.01541461	0.252719677
NC-12	0.41403069	0.04142774	0.04037939	0.02573474	0.100059591	0.09752754	0.06215660	0.159684134	0.04738105	0.01317606	0.278087093
NC-16	0.40630188	0.03929329	0.04099477	0.02177997	0.096709586	0.10089733	0.05360538	0.15450271	0.03777216	0.01843124	0.487958393
NC-20	0.40089132	0.04016594	0.04150062	0.02504198	0.100191602	0.10352088	0.06246576	0.165986646	0.06198106	0.01682471	0.271449178
NC-24	0.37190507	0.03657216	0.03949652	0.02485740	0.098337357	0.10620053	0.06683803	0.17303856	0.06711784	0.01853712	0.276187649
NC-28	0.43542446	0.04621624	0.04749900	0.02324652	0.106140653	0.10908667	0.05338817	0.162474841	0.05165213	0.01486954	0.287878551
NC-32	0.39342040	0.03449628	0.03976731	0.01663950	0.087683008	0.10108096	0.04229445	0.143375413	0.05375858	0.01263181	0.234972854

**Table 2:** Data on content of DA and DA metabolites (DOPAC, HVA, 3-MT), contents of 5-HT and the 5-HT metabolite (5-HIAA), DA turnover (DOPAC/DA, HVA/DA, 3-MT/DA, (HVA+3-MT)/DA), and 5-HT turnover (5-HIAA/5-HT) in the hypothalamus of the male offspring mice given tap water or 125, 250, or 500 ppm PTU solution from GD15 to PND25. Medians, 1st quartiles and 3rd quartiles of these data are shown in Fig. 4. Unit of data is nmol/mg protein.

Sample Name	Dopamine	DOPAC	HVA	3MT	DOPAC/DA	HVA/DA	3-MT/DA	(HVA+3MT)/DA	5-HT	5-HIAA	5HIAA/5HT
Tap Water											
HT-1	0.03007494	0.00681148	0.00851213	0.00041095	0.226483666	0.283030467	0.013664066	0.296694533	0.07900271	0.01995802	0.252624441
HT-5	0.036391724	0.00920114	0.013983465	0.00139750	0.252836072	0.384248484	0.038401533	0.422650017	0.07531993	0.02970364	0.394366302
HT-9	0.04279445	0.01157502	0.01831978	0.00244641	0.270479478	0.428087718	0.057166452	0.485254170	0.08333999	0.03260346	0.391210263
HT-13	0.037027811	0.00924903	0.012981616	0.00352508	0.249786024	0.350590954	0.095200805	0.445791759	0.082369479	0.02839823	0.344766357
HT-17	0.04734070	0.01158008	0.01787977	0.00279289	0.244611467	0.377682788	0.058995643	0.436678431	0.08472127	0.02972774	0.350888686
HT-21	0.04125577	0.01077268	0.01764479	0.00370809	0.261119337	0.427692706	0.089880526	0.517573232	0.08480397	0.03361082	0.396335415
HT-25	0.04268676	0.01190796	0.01664119	0.00317740	0.278961366	0.389844361	0.074435203	0.464279564	0.07649257	0.02718259	0.355362518
HT-29	0.04039764	0.01095842	0.01790328	0.00306500	0.271263889	0.443176411	0.075870734	0.519047145	0.08414374	0.02974272	0.353475112
PTU125PPM											
HT-2	0.05511178	0.01280188	0.01901535	0.00521091	0.232289290	0.345032356	0.094551718	0.439584074	0.08521873	0.02585196	0.303360024
HT-6	0.04645041	0.01281198	0.01440925	0.00207864	0.275820615	0.31020717	0.044749647	0.354956817	0.08577008	0.03153575	0.367677698
HT-10	0.04061807	0.01252799	0.01952043	0.00378521	0.308433784	0.48058477	0.093190181	0.573774951	0.08132756	0.02833154	0.348363394
HT-14	0.04624095	0.01501858	0.01531263	0.00422935	0.324789575	0.331148779	0.09146337	0.422612149	0.09772496	0.03722352	0.380900851
HT-18	0.05526921	0.01485856	0.01648089	0.00422436	0.268839694	0.298192895	0.07643250	0.374625390	0.07824131	0.02634782	0.336750801
HT-22	0.04055246	0.01092303	0.01375298	0.00233086	0.269355526	0.339140512	0.057477576	0.396618088	0.07832751	0.02631086	0.335908277
HT-26	0.03658542	0.00995936	0.01356472	0.00000000	0.272222189	0.370768414	0	0.370768414	0.07950551	0.02557869	0.32172229
HT-30	0.04118250	0.01283607	0.01302857	0.00000000	0.311687482	0.316361759	0	0.316361759	0.07994540	0.02905260	0.36340546
PTU250PPM											
HT-3	0.04210345	0.01305596	0.02036711	0.00473362	0.310092441	0.48373966	0.112428365	0.596168028	0.08134562	0.02658578	0.326824945
HT-7	0.04059358	0.01201760	0.01817081	0.00000000	0.296046776	0.44762770	0	0.447627696	0.07661086	0.02897390	0.378195675
HT-11	0.03872511	0.01020422	0.01417830	0.00000000	0.263504105	0.36612684	0	0.366126842	0.07724246	0.02717619	0.351829612
HT-15	0.05468781	0.01293366	0.02185233	0.00448399	0.236499887	0.39958324	0.081992476	0.481575711	0.08054023	0.02390253	0.296777559
HT-19	0.04417410	0.01106164	0.01468611	0.00161018	0.250409998	0.33245981	0.036450853	0.368910659	0.08013623	0.02350880	0.293360412
HT-23	0.03995035	0.01116150	0.01711718	0.00313612	0.279384432	0.42846146	0.078500466	0.506961924	0.08279249	0.02870700	0.346734346
HT-27	0.04352635	0.01063112	0.01362185	0.00241226	0.244245582	0.31295643	0.05542080	0.368377223	0.07527058	0.02410385	0.320229401
HT-31	0.03827161	0.01034299	0.01514347	0.00268201	0.270252268	0.39568407	0.07007820	0.465762275	0.07472096	0.02090493	0.279773332
PTU500PPM											
HT-4	0.04445283	0.01299876	0.01512903	0.00000000	0.292416908	0.34033889	0.00000000	0.340338889	0.08319888	0.03056792	0.367407871
HT-8	0.04596222	0.01079323	0.01358984	0.00588573	0.234828225	0.29567410	0.12805576	0.423729855	0.09147560	0.02488435	0.272032643
HT-12	0.03994688	0.00881913	0.01314792	0.00000000	0.220771398	0.32913512	0.00000000	0.329135115	0.07877459	0.02213291	0.280965061
HT-16	0.04073818	0.01070071	0.01561375	0.00333812	0.262670180	0.38327053	0.08194072	0.465211257	0.08918300	0.02847222	0.319256161
HT-20	0.04422160	0.00962038	0.01356376	0.00175995	0.217549339	0.30672259	0.03979853	0.346521125	0.09942045	0.03140696	0.315900353
HT-24	0.03808130	0.00935550	0.01271361	0.00248157	0.245671845	0.33385439	0.06516501	0.399019403	0.07888817	0.02734967	0.346689102
HT-28	0.05943981	0.01547961	0.02172213	0.00422753	0.260424989	0.36544747	0.07112283	0.436570293	0.08520443	0.02859538	0.335609036
HT-32	0.03186199	0.00743416	0.01088377	0.00224627	0.233323896	0.34159099	0.07050010	0.412091091	0.06842596	0.01827524	0.267080503

**Table 3:** Data on content of DA and DA metabolites (DOPAC, HVA, 3-MT), contents of 5-HT and the 5-HT metabolite (5-HIAA), DA turnover (DOPAC/DA, HVA/DA, 3-MT/DA, (HVA+3-MT)/DA), and 5-HT turnover (5-HIAA/5-HT) in the hippocampus of the male offspring mice given tap water or 125, 250, or 500 ppm PTU solution from GD15 to PND25. Medians, 1st quartiles and 3rd quartiles of these data are shown in Fig. 5. Unit of data is nmol/mg protein.

Sample Name	Dopamine	DOPAC	HVA	3MT	DOPAC/DA	HVA/DA	3-MT/DA	(HVA+3MT)/DA	5-HT	5-HIAA	5HIAA/5HT
Tap Water											
HP-1	0.00000000	0.00166201	0.00285147	0.00000000					0.04949900	0.01959967	0.395960993
HP-5	0.007845654	0.003134367	0.005501158	0.00000000	0.399503613	0.701172631	0	0.701172631	0.04229350	0.02688851	0.635759864
HP-9	0.00000000	0.00200885	0.00298120	0.00000000					0.05410971	0.03589520	0.663378185
HP-13	0.00362840	0.001452932	0.002329013	0.00132645	0.400433638	0.64188517	0.365575078	1.007460248	0.042009985	0.02422860	0.576734369
HP-17	0.00149163	0.00228745	0.00434343	0.00000000	1.533521060	2.911865404	0	2.911865404	0.05619225	0.03220269	0.573080617
HP-21	0.00317025	0.00219362	0.00432921	0.00000000	0.691938155	1.365573419	0	1.365573419	0.04951699	0.03830863	0.773646190
HP-25	0.00417028	0.00239853	0.00279441	0.00635504	0.575148128	0.670075722	1.523887481	2.193963203	0.05125057	0.02942192	0.574079840
HP-29	0.00518678	0.00249031	0.00302974	0.00000000	0.480126193	0.584127718	0	0.584127718	0.05089126	0.03071904	0.603621144
PTU125PPM											
HP-2	0.00238557	0.00221824	0.00656487	0.00000000	0.929855844	2.751903535	0	2.751903535	0.06039198	0.02817683	0.466565768
HP-6	0.00228949	0.00204994	0.00340209	0.00000000	0.895369326	1.485960158	0	1.485960158	0.04639133	0.03168330	0.682957424
HP-10	0.00000000	0.00301943	0.00335039	0.00000000					0.05449218	0.03076623	0.564599057
HP-14	0.00301981	0.00166575	0.00152167	0.00099482	0.551606270	0.50389550	0.329430893	0.833326398	0.05955821	0.03113127	0.522703171
HP-18	0.00155129	0.00297082	0.00443379	0.00000000	1.915056282	2.858121514	0.00000000	2.858121514	0.04844648	0.03273393	0.675671900
HP-22	0.00333919	0.00253754	0.00405752	0.00000000	0.759926111	1.215122463	0	1.215122463	0.05830162	0.03067086	0.526072179
HP-26	0.00000000	0.00350942	0.00355928	0.00000000					0.06303965	0.03287718	0.521531829
HP-30	0.00204007	0.00346474	0.00370452	0.00000000	1.698340791	1.815876434	0	1.815876434	0.05373357	0.02953119	0.549585400
PTU250PPM											
HP-3	0.00000000	0.00162638	0.00483846	0.00000000					0.05443471	0.03034676	0.557489227
HP-7	0.00000000	0.00157311	0.00378837	0.00000000					0.04745613	0.02754890	0.580512975
HP-11	0.00000000	0.00184601	0.00296020	0.00000000					0.05257766	0.02916047	0.554617219
HP-15	0.00269084	0.00197063	0.00514580	0.00000000	0.732348616	1.91234091	0	1.912340909	0.05946732	0.03182276	0.535130170
HP-19	0.00083547	0.00250855	0.00348978	0.00000000	3.002576277	4.17703901	0	4.177039015	0.05677314	0.02591757	0.456511142
HP-23	0.00000000	0.00161200	0.00396172	0.00000000					0.05102750	0.02771402	0.543119152
HP-27	0.00348227	0.00227959	0.00625176	0.00594401	0.654627236	1.79531042	1.70693587	3.502246291	0.04616985	0.02203186	0.477191515
HP-31	0.00262972	0.00379306	0.00396464	0.00000000	1.442379865	1.50762614	0.00000000	1.507626136	0.05893444	0.02237122	0.379594951
PTU500PPM											
HP-4	0.00000000	0.00226449	0.00185804	0.00000000					0.05085629	0.02766179	0.543920633
HP-8	0.00740318	0.00412270	0.00592177	0.00579696	0.556882031	0.79989530	0.78303677	1.582932076	0.05769458	0.02488137	0.431260185
HP-12	0.00357564	0.00148444	0.00222236	0.00112793	0.415153467	0.62152650	0.31544899	0.93697549	0.04530969	0.02171460	0.479248565
HP-16	0.00000000	0.00221568	0.00361642	0.00000000					0.06672642	0.02677478	0.401262069
HP-20	0.00412490	0.00243529	0.00299414	0.00000000	0.590386566	0.72586942	0.00000000	0.725869418	0.06223070	0.03567057	0.573198935
HP-24	0.00149883	0.00296618	0.00481829	0.00000000	1.978995517	3.21469905	0.00000000	3.21469905	0.06447874	0.03991882	0.619100525
HP-28	0.00280867	0.00308508	0.01956428	0.00000000	1.098414358	6.96567558	0.00000000	6.965675582	0.05699487	0.02978307	0.522557047
HP-32	0.00415735	0.00058783	0.00381481	0.00000000	0.141394711	0.91760670	0.00000000	0.917606704	0.05235606	0.02310756	0.441354033

## 2 Experimental design, materials, and methods

### 2.1 Animals

ICR (or CD-1) strain mice were used.

### 2.2 Drug

Propylthiouracil (6-Propyl-2-thiouracil or 2,3-Dihydro-6-propyl-2-thioxo-4(1H)-pyrimidinone; PTU) solution at concentrations of 125, 250, or 500 ppm was given as drinking water to pregnant females.

### 2.3 Experimental procedures

#### 2.3.1 Perinatal exposure to PTU, measurement of growth, and observation of eye opening in offspring mice

The day when a plug was observed in the dam was defined as GD0. Administration of 125, 250, or 500 ppm PTU solution was started on GD15. Tap water was given to control dams. The solution was available *ad libitum* during the exposure period. The day when delivery was observed was defined as PND0. On PND0, body weight of offspring were measured, followed by measuring the body weight and checking eye opening of offspring every 3–4 days. The offspring mice were weaned, male and female offspring were separated, and exposure to PTU was terminated on PND25. Tap water was given to all offspring mice thereafter. On PND25, the body weight of offspring was measured, followed by measurement of the body weight every 3–4 days.

#### 2.3.2 Blood collection for the serum T4 assay

On PND25, blood collection for the serum T4 measurements was performed. Blood was transcardially collected under deep anesthesia form dams and one or two male and female offspring randomly selected from each litter. The serum samples of the collected blood were stored at −80 °C and subjected to T4 measurement at a later time.

#### 2.3.3 Brain monoamine content measurement

The male offspring mice were sacrificed by decapitation on PND60, and the brain was removed. The brain was placed on ice, and the nucleus accumbens, hypothalamus, and hippocampus were bilaterally collected, and frozen in liquid nitrogen. The tissue were kept at −135 °C and later subjected to the monoamine content measurement.

#### 2.3.4 Serum T4 assay

Serum T4 levels in the collected blood samples were measured by radioimmunoassay using DPC・total T4 kits (Yatoron Co., Ltd. (present: LSI Medience Co., Ltd.), Tokyo, Japan) according to the company's instructions.

#### 2.3.5 Brain monoamine contents measurement

Samples of the nucleus accumbens, hypothalamus and hippocampus were sonicated in 0.4 N perchloric acid, followed by centrifugation at 17,760×*g* for 15 min at 4 °C. DA, DOPAC, HVA, 3-MT, 5-HT, and 5-HIAA levels in the supernatant samples were measured using high performance liquid chromatography with electrochemical detection (HPLC/ECD) system (L-5000; Yanaco, Kyoto, Japan). Chromatographic separation was made using a 4.6 mm × 250 mm ODS-C18 column (Nacalai, Kyoto, Japan) at 19 °C.The flow rate of the mobile phase (0.1 M citric acid, 15% MeOH, 0.1 mM octane sulfonic acid, and 0.1 mM Na_2_EDTA adjusted to pH 2.5) was 1.0 mL/min. Monoamines were measured using an electrochemical detector (VMD-101A; Yanaco) with a glassy carbon electrode. The applied voltage was +750 mV against an Ag/AgCl reference electrode. Protein concentration was measured using a BCA protein assay kit (Pierce, Rockford, IL, USA) according to the company's instruction.

### 2.4 Statistical analyses

Changes in body weight were analyzed using repeated measures analysis of variance (ANOVA), followed by one-way ANOVA and Dunnett's test for each PND. The day of eye opening, monoaminergic neurotransmitter contents, metabolite contents, and turnover in the brain regions were analyzed using the Wilcoxon test. P < 0.05 was considered as statistically significant.
